# Supplementary material for: The causal role of circulating inflammatory markers in osteoporosis: a bidirectional Mendelian randomized study
Source: Front Immunol. 2024 Jul 18;15:1412298. doi: 10.3389/fimmu.2024.1412298 (PMC11291241; doi:10.3389/fimmu.2024.1412298)
Supplement: Supplementary file 1 [file DataSheet_1.zip › supplementary material/OSTEOPOROSIS-CIM/GCST90274820.tsv.gz_forest.pdf]

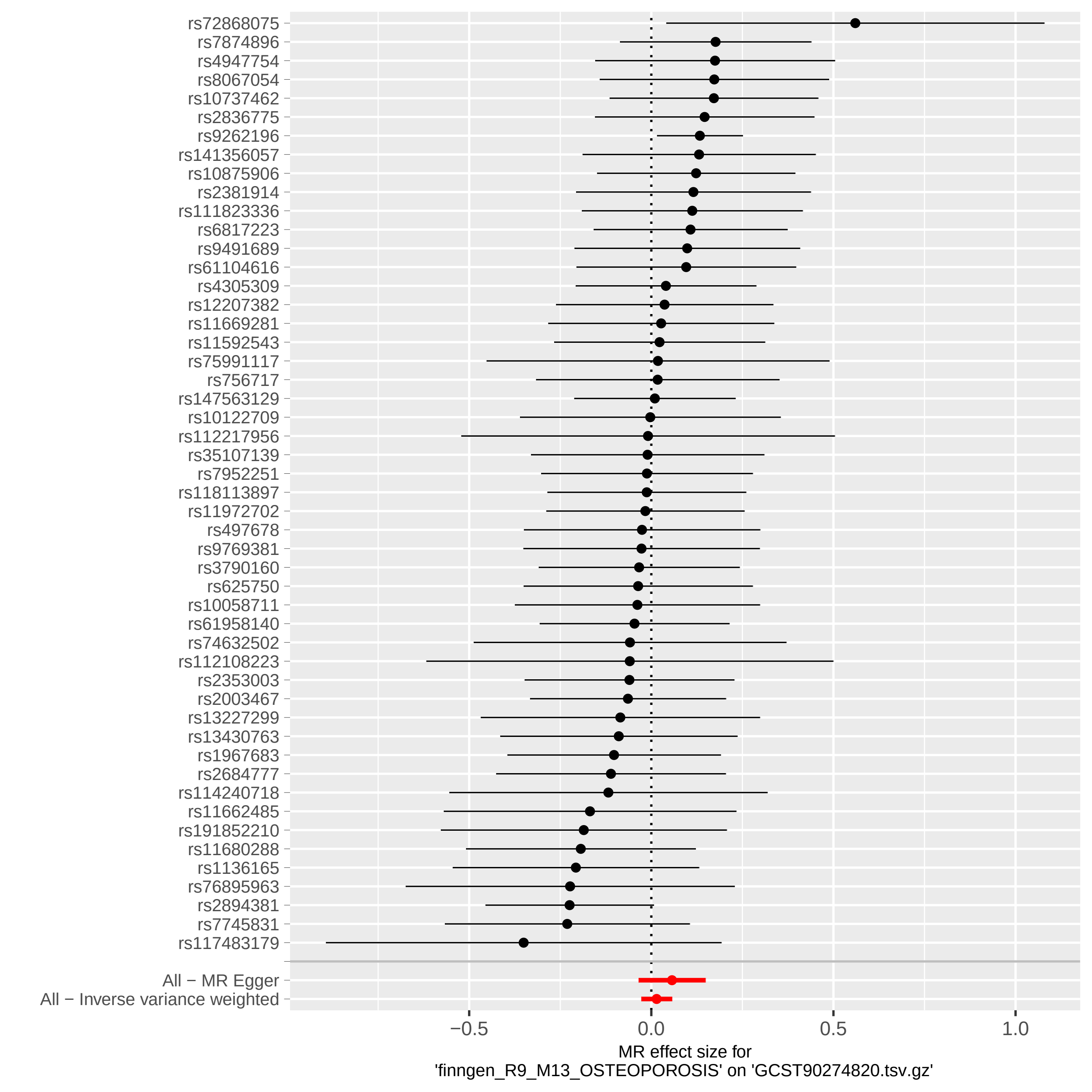

rs72868075
rs7874896
rs4947754
rs8067054
rs10737462
rs2836775
rs9262196
rs141356057
rs10875906
rs2381914
rs111823336
rs6817223
rs9491689
rs61104616
rs4305309
rs12207382
rs11669281
rs11592543
rs75991117
rs756717
rs147563129
rs10122709
rs112217956
rs35107139
rs7952251
rs118113897
rs11972702
rs497678
rs9769381
rs3790160
rs625750
rs10058711
rs61958140
rs74632502
rs112108223
rs2353003
rs2003467
rs13227299
rs13430763
rs1967683
rs2684777
rs114240718
rs11662485
rs191852210
rs11680288
rs1136165
rs76895963
rs2894381
rs7745831
rs117483179
All − MR Egger
All − Inverse variance weighted
−0.5
0.0
0.5
1.0
MR effect size for
'finngen_R9_M13_OSTEOPOROSIS' on 'GCST90274820.tsv.gz'
